# E2F2 directly regulates the STAT1 and PI3K/AKT/NF-κB pathways to exacerbate the inflammatory phenotype in rheumatoid arthritis synovial fibroblasts and mouse embryonic fibroblasts

**DOI:** 10.1186/s13075-018-1713-x

**Published:** 2018-10-04

**Authors:** Shiguan Wang, Lin Wang, Changshun Wu, Shui Sun, Ji-hong Pan

**Affiliations:** 1 Medical and Life Science College, University of Jinan, Jinan, 250062 Shandong China; 2 Shandong Medicinal Biotechnology Centre, Jingshi Road, Jinan, 250000 Shandong China; 3 Key Lab for Biotechnology Drugs of Ministry of Health, Jinan, 250000 Shandong China; 4 Key Lab for Rare & Uncommon Diseases, Jinan, 250000 Shandong China; 5 Shandong Provincial Hospital affiliated to Shandong University, Jinan, 250000 Shandong China

**Keywords:** Cytokine, Inflammation, Rheumatoid arthritis, Synovial fibroblast, E2F2

## Abstract

**Background:**

Expression of E2F transcription factor 2 (E2F2), a transcription factor related to the cell cycle, is abnormally high in rheumatoid arthritis synovial fibroblasts (RASFs). Deregulated expression of E2F2 leads to abnormal production of proinflammatory cytokines, such as interleukin (IL)-1α, IL-1β, and tumor necrosis factor (TNF)-α in RASFs. However, the underlying mechanism by which E2F2 regulates expression of IL-1α, IL-1β, and TNF-α has not been fully elucidated. This study aimed to elucidate this mechanism and confirm the pathological roles of E2F2 in rheumatoid arthritis (RA).

**Methods:**

*E2f2* knockout (KO) and wild-type (WT) mice were injected with collagen to induce RA. Cytokine production was assessed by quantitative real-time polymerase chain reaction (qRT-PCR) and enzyme-linked immunosorbent assay (ELISA). Western blot and qRT-PCR were performed to evaluate the effect of E2F2 on signaling pathway activity. Chromatin immunoprecipitation (ChIP)-PCR and luciferase assays were used to detect the transcriptional activity of target genes of E2F2. Nuclear translocation of STAT1 and p65 were assayed by Western blot, co-immunoprecipitation (co-IP), and immunofluorescence experiments.

**Results:**

The occurrence and severity of collagen-induced arthritis were decreased in *E2f2*-KO mice compared with WT mice. The expression of IL-1α, IL-1β, and TNF-α was also suppressed in mouse embryonic fibroblasts (MEFs) from *E2f2*-KO mice and RASFs with E2F2 knocked down. Mechanistically, we found that E2F2 can upregulate the expression of STAT1 and MyD88 through direct binding to their promoters, facilitate the formation of STAT1/MyD88 complexes, and consequently activate AKT. However, silencing STAT1/MyD88 or inactivating AKT significantly attenuated the induction of IL-1α, IL-1β, and TNF-α caused by the introduction of E2F2.

**Conclusions:**

This study confirms the pathological role of E2F2 in RA and found that the E2F2-STAT1/MyD88-Akt axis is closely related with the inflammatory phenotype in RASFs.

## Background

Rheumatoid arthritis (RA) is a systemic autoimmune disease resulting in severe inflammation and morphological changes to skeletal tissues, including bone and joint damage. Cartilage degradation, synovial hyperplasia, fibroblast-like synoviocyte infiltration into cartilage and bone surfaces, and subchondral bone erosion are all pathological hallmarks of RA [1]. Accumulating evidence suggests that activated rheumatoid arthritis synovial fibroblasts (RASFs) play an important role in the pathogenesis of arthritic joint destruction [2, 3]. The process of arthritic joint destruction results in a marked secretion of inflammatory cytokines, including interleukin (IL)-1α, IL-1β, and tumor necrosis factor (TNF)-α, which are important in inducing adaptive immunity in RASFs [4]. In RA, excessive inflammatory molecules such as IL-1α, IL-1β, and TNF-α are secreted and subsequently mediate the destruction of cartilage and bone [5]. The identification of pathogenic genes regulating inflammation in the microenvironment of the inflamed joint is crucial to understand the pathogenesis of RA and may also provide new targets for diagnosis and treatment.

E2F transcription factor 2 (E2F2) encodes a periodic cycling molecule involved in transcription factor activity, sequence-specific DNA binding, and core promoter binding. Inhibition of E2F expression by an E2F decoy oligonucleotide inhibits the proliferation of synovial cells and prevents cartilage invasion [6]. A microarray study of peripheral blood mononuclear cells from RA patients found that a significant number of RA-associated genes contain E2F-binding motifs in their promoters, suggesting that E2F2 may play a role in RA pathogenesis [7–9].

In our previous microarray analysis, E2F2 was highly expressed in RA synovial tissue [10]. In RASFs, activated NF-κB can bind to the promoter region of E2F2, and activated E2F2 can bind to the promoter of IL-6 which, in turn, can promote the progression of arthritis [7]. Our recent data further showed that expression of E2F2 can promote upregulation of IL-1α, IL-1β, and TNF-α, although the mechanism underlying this function remains to be discovered. In the present study, RASFs and a gene knockout (KO) mouse model were used to investigate the signaling pathways involved in the pathogenic role of E2F2 in RA.

## Methods

### Cell acquisition and arthritis models

Synovial tissues were collected during knee joint replacement surgery from patients with RA (*n* = 14; five males, nine females, aged 35 to 75 years old, mean age 55 years). All patients fulfilled the 1987 American College of Rheumatology revised criteria for RA diagnosis. Written informed consent was obtained from each patient, and all samples were rendered anonymous. The Ethical Committee of the Shandong Academy of Medicinal Sciences approved this study (approval number 2014–2019).

### E2F2 knockout mice

Twenty transgenic founder (F0) *E2f2*^*+/−*^ mice (C57Bl6:129Sv background, 11 females, 9 males) from The Jackson Laboratory (JAX, Bar Harbor, Maine, USA) were identified using polymerase chain reaction (PCR) with genomic DNA. Female and male transgenic F0 mice were mated to produce the F1 generation. Tail clips were dissected from F1 mice at postnatal day 12 and subjected to PCR to determine *E2f2* expression. Eight-week-old male and female mice (*E2f2*^*+/−*^) were mated to obtain *E2f2*^*−/−*^ and wild-type (WT) mice. These mice were maintained on a normal light/dark cycle in cages with microisolator lids, and genotyped by standard PCR [11, 12]. All procedures were approved by the Animal Care and Use Committee of the Shandong Academy of Medical Sciences.

### Collagen-induced arthritis (CIA) model

CIA is a well-established mouse model for human RA [13]. Arthritic mice develop swollen joints, chronic inflammation, and joint destruction. CIA was induced by injecting a type II collagen at 2 mg/mL (Chondrex, Washington, USA) and complete Freud’s adjuvant (Sigma-Aldrich, Mannheim, Germany) 1:1 emulsion (200 μL) at the base of the tail in 10-week-old male WT and *E2f2*^−/−^ mice. A type II collagen and incomplete Freud’s adjuvant (Sigma-Aldrich) 1:1 emulsion was injected (200 μL) at the base of the tail in 14-week-old mice. Mice were monitored once per day for symptoms of arthritis.

### Mouse embryonic fibroblast (MEF) cell culture

*E2f2*^*−/−*^ and wild-type MEFs were obtained from *E2f2*^*+/−*^ maternal embryos at 13.5 days gestation as previously described [12]. The extracted MEFs were cultured in Dulbecco’s modified Eagle’s medium (DMEM) high glucose medium containing 10% serum and 1% penicillin/streptomycin.

### Stimulation assays

Primary RASFs were isolated and cultured as described previously [14], and cells were used between passages 3 and 7. We screened many commonly used inflammatory stimulators including IL-1β, TNF-α, interferon (IFN)α, lipopolysaccharide (LPS), and IL-6 to stimulate RASF and MEFs, but we found LPS to be the most efficient. RASFs were plated in 24-well plates (3–5 × 10^5^ cells/well) and stimulated for 12 h with LPS (from *Escherichia coli* J5; Sigma, St Louis, MO, USA). The dilution buffer, phosphate-buffered saline (PBS), was applied as a control. Activation of PI3K/AKT pathways was blocked using LY294002 (Calbiochem, MCE, New Jersey, USA) and NF-κB inhibitor PDTC (M4005, Abmole Bioscience, Hong Kong, China).

### Luciferase reporter gene assay

HEK239T cells (2 × 10^4^ cells/well; 96-well plate) were seeded in triplicate in 24-well plates and transfected with 80 ng/well STAT1 and MyD88 luciferase reporter plasmids using Lipofectamine 2000, as described by the manufacturer (Invitrogen, Carlsbad, CA, USA). The reporter gene plasmids used were: pGL4.10-STAT1 promoter-WT; pGL4.10-STAT1 promoter-mutant (mut); pcDNA3.1(+)-E2F2; pRL-CMV (control) and pGL4.10-MyD88 promoter-WT; pcDNA3.1(+)-E2F2; and pRL-CMV (control). All plasmids were constructed by Obio (Obio, Shanghai, China). In all cases, 40 ng/well of phRL-TK reporter gene activity was measured using the Dual Luciferase Assay system (Promega, Madison, Wisconsin, USA). Data are expressed as the mean fold induction ± SEM relative to control levels from a minimum of three separate experiments.

### Small interfering RNA (siRNA) and adenovirus transfection in RASFs and MEFs

RASFs (2 × 10^5^ cells in 100-mm diameter dishes or 8 × 10^4^ cells in six-well plates) were transiently transfected with siRNA targeting E2F2 (SI00375410, Qiagen, Hilden, Germany) or negative control siRNA (1,027,281, Qiagen) using HiPerFect transfection reagent (Qiagen) following the manufacturer’s instructions, and all experiments were performed 24 h after transfection. The specific siRNAs targeting STAT1 were designed and synthesized by RuiboBio (RuiboBio, Guangzhou, China), and the most effective single siRNA was used for further experiments as follows: STAT1 (homo): CCTACGAACATGACCCTAT; STAT1 (mus): CTGTGATGTTAGATAAACA; MyD88 (homo): CCATCAAGTACAAGGCAAT. RASFs (2 × 10^5^ cells in 100-mm diameter dishes or 8 × 10^4^ cells in six-well plates) were infected by E2F2 and STAT1 adenovirus (2 × 10^5^ pfu/mL) or empty adenovirus (ViGeneBiosciences, JiNan, China) using ADV-HR (ViGeneBiosciences) following the manufacturer’s instructions. MEFs (2 × 10^5^ cells in 100-mm diameter dishes or 8 × 10^4^ cells in six-well plates) were infected by STAT1 adenovirus (2 × 10^5^ pfu/mL) or empty adenovirus (Obio, Shanghai, China) using ADV-HR (Obio) following the manufacturer’s instructions.

### Enzyme-linked immunosorbent assay (ELISA)

Cells were cultured and stimulated as described above, and supernatants were collected at 12 h. The release of IL-1α, IL-1β, and TNF-α was analyzed by ELISA (MultiSciences, Hang Zhou, China) according to the manufacturer’s instructions. Serum was extracted from fresh blood in 20-week-old mice. After centrifugation to remove particulates, the release of IL-1α, IL-1β, and TNF-α was analyzed by ELISA (MultiSciences) according to the manufacturer’s instructions.

### Western blot

Whole cell lysates were separated by sodium dodecyl sulfate-polyacrylamide gel electrophoresis (SDS-PAGE) and transferred onto a 0.45-μm Immobilon-P transfer membrane (Merck Millipore, Darmstadt, Germany). They then underwent immunoblotting with the following specific primary antibodies overnight at 4 °C: anti-E2F2 (1:1000, Millipore), anti-STAT1 (1:1000, CST, Boston, MA, USA), anti-AKT (1:1000, CST), phospho-AKT (1:1000, CST), phospho-p65 NF-κB (1:1000, Affinity, Cincinnati, OH, USA) or p65 NF-κB (1:1000, Affinity), and anti-MyD88 (1:1000, Affinity). After washing with TBST, the membrane was incubated with each corresponding secondary antibody for 1 h at 37 °C. Detection was performed using an ECL Plus detection system (Thermo Scientific, Pittsburgh, PA, USA).

### Co-immunoprecipitation (co-IP) assays

Immunoprecipitation was carried out to assess the interaction between STAT1 and MyD88. After harvesting the RASFs and MEFs, the supernatants were incubated overnight at 4 °C with rabbit anti-STAT1 (1:200, CST) and then protein A/G-Sepharose beads (Beyotime, Suzhou, China) conjugated to STAT1. The samples were then electrophoresed through gradient SDS-polyacrylamide gels and transferred to membranes that were probed with mouse anti-STAT1 (1:1000, Proteintech, Wuhan, China). Following incubation with horseradish peroxidase-conjugated secondary antibodies (458, MBL, Tokyo, Japan) the blots were developed using an ECL Plus detection system (Thermo Scientific).

### RNA extraction and quantitative real-time PCR (qRT-PCR)

Total RNA was extracted from cultured cells and human tissues using TRIzol Reagent (Invitrogen) according to the manufacturer’s protocol. RNA was reverse-transcribed using a ReverTra Ace qPCR RT Kit (Toyobo, Tokyo, Japan). qRT-PCR was conducted using a LightCycler 480 (Roche, Basel, Switzerland) with the following protocol: denaturation at 95 °C for 10 min, 40 cycles of denaturation at 95 °C for 10 s, annealing at 60 °C for 1 min, and extension at 72 °C for 1 s. The forward and reverse primers are shown in Table 1. All primers were synthesized by BGI (Beijing, China). Each sample was analyzed in triplicate. The 2^–△△Ct^ method of relative quantification was used to calculate changes in the expression of target genes.

**Table 1:** Primers used for real-time polymerase chain reaction

Primer name	Primer base sequence (5’ to 3’)
GAPDH (homo)	Forward: CACCATCT TCCAGGAGC;Reverse: AGTGGACTCCACGACGTA
GAPDH (mus)	Forward: AAAGGGTCATCATCTCCG;Reverse: CAATCTTGAGTGAGTTGTCATATTTC
E2F2 (homo)	Forward: CCTTGGA GGCTACTGACAGC;Reverse: CCACAGGTAGTCGTCCTGGT
E2F2 (mus)	Forward: TGTTTCCCTGGGAGGATTATT;Reverse: TTTGGGACAGTGGGTGTTTA
STAT1 (homo)	Forward: TACACCTACGAACATGACCC;Reverse: TGAAGGTGCGGTCCCATAA
STAT1 (mus)	Forward: TGGGAAGTATTATTCCAGACCAAA;Reverse: AGTCTTGATGTATCCAGTTCG
MyD88 (homo)	Forward: CTGGCCTCTGGCATATTC;Reverse: CTCCCTGCTCACATCATTAC
MyD88 (mus)	Forward: TGCCAGCGAGCTAATTG;Reverse: CACATTCCTTGCTCTGTAGATA
AKT1 (homo)	Forward: GGCGTGGTCATGTACGA;Reverse: TTCTCATGGTCCTGGTTGTAG
AKT1 (mus)	Forward: GGACGGGCACATCAAGATAA;Reverse: CCGCAGAATGTCTTCATAG
AKT2 (homo)	Forward: TACACCTACGAACATGACCC;Reverse: TGAAGGTGCGGTCCCATAA
AKT2 (mus)	Forward: TTCAGAAGTGGACACAAGGT;Reverse: GGGTCCAGGCTGTCATATC
IL-1α (homo)	Forward: CGTCAGGCAGAAGTTTGTCA;Reverse: TTAGAGTCGTCTCCTCCCGA
IL-1α (mus)	Forward: ATCACAGGTAGTGAGACCGA;Reverse: AGCTGATGTGAAGTAGTTCTTAG
IL-1β (homo)	Forward: CTAAAGTATGGGCTGGACTG;Reverse: AGCTTCAATGAAAGACCTCA
IL-1β (mus)	Forward: CAAGGAGAACCAAGCAACGA;Reverse: TTTCATTACACAGGACAGGTATAGA
TNF-α (homo)	Forward: TGTCTACTGAACTTCGGGGT;Reverse: TCACAGAGCAATGACTCCAA
TNF-α (mus)	Forward: AGGTTCTCTTCAAGGGACAA;Reverse: GACTTTCTCCTGGTATGAGATAG

*IL* interleukin, *TNF* tumor necrosis factor

### Chromatin immunoprecipitation (ChIP)-PCR

RASFs and MEFs were treated with LPS for 12 h and then fixed with 1% formaldehyde for 10 min at room temperature. DNA was broken into 200–1000 bp fragments using a sonicator (10^6^ cells in 200 μL volume, ultrasound 10 s, stop 10 s, repeated seven times). Chromatin was immunoprecipitated with immunoglobulin (Ig)G (Sigma) and anti-E2F2 (Millipore). The association of E2F2 with STAT1 and MyD88 was measured by RT-PCR (predenaturation at 95 °C for 5 min, followed by 95 °C for 30 s, 65 °C for 30 s, and 72 °C for 30 s, 35 cycles) using immunoprecipitated chromatin from RASFs with the following primers: STAT1: 5′- TGCATAGGGCTCAGGCA -3′ (forward) and 5′- CCCTTAGCCTCTTTCTGTTC -3′ (reverse) and 5′- TGAGGTAGGTAGGCCCTT -3′ (forward) and 5′-TCTTAGGGTGAACTCGGCA -3′ (reverse); MyD88: 5′- CTAAATACTTCCGAGACGCC -3′ (forward) and 5′- CAGTTAGAGAGCTTGTCACAC -3′ (reverse).

### Immunofluorescence microscopy

MEFs and RASFs were grown in 48-well plates with LPS stimulation for 12 h. Preconditioned cells were washed three times with PBS slowly for 3 min each and then fixed with 4% paraformaldehyde for 10 min, washed three times with PBS (3 min each) and then treated with 5% bovine serum albumin (BSA) for 1 h. Cells were then incubated with monoclonal anti-MyD88 (1:100) and polyclonal anti-STAT1 (1:100) antibodies overnight at 4 °C. After three rinses, FITC- and TRITC-conjugated secondary antibodies were used to visualize the proteins by fluorescence microscopy (Olympus Corporation, FV3000, Tokyo, Japan). The nuclei were stained with 4’,6’-diamidino-2-phenylinndole (DAPI).

### Histological analysis

Paws were fixed for 24 h in 10% buffered formalin and decalcified in 15% ethylenediaminetetraacetic acid (EDTA). The paws were then embedded in paraffin, and serial 5-μm sagittal sections were cut and stained with hematoxylin and eosin (H&E). Sections were also stained with Safranin O-Fast Green to determine the depletion of proteoglycans.

### Statistical analysis

Statistical analysis was performed using the GraphPad Prism 5 software package (La Jolla, CA, USA). Results were considered statistically significant at *P* < 0.05. The results are expressed as mean ± SEM of five different experiments. The data were analyzed by two-way analysis of variance (ANOVA) followed by Bonferroni’s multiple comparison test. The statistical significance of differences in the central tendencies were designated as *P < 0.05, **P < 0.01, and ***P < 0.001.

## Results

### Amelioration of the CIA inflammatory phenotype in *E2f2* KO mice

**Fig. 1:**
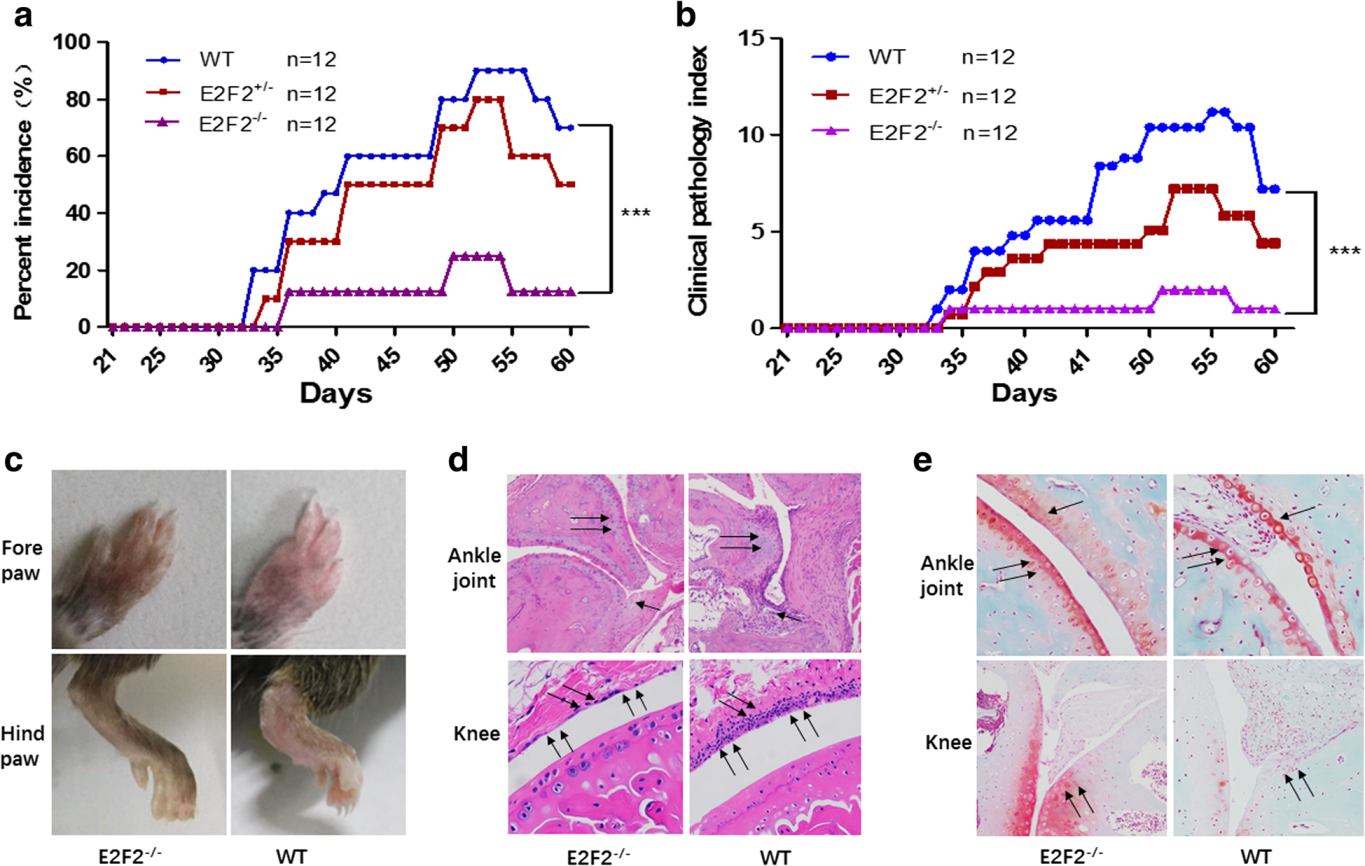
E2F2 affects the incidence and degree of CIA in mice. Arthritis was induced in wild-type (WT), *E2f2*^*+/−*^, and *E2f2*^*−/−*^ mice and the incidence (**a**) and clinical pathology (**b**) of arthritis were evaluated as 0 (no swelling) to 4 (strong swelling) once per day by two independent observers under blinded conditions. All results are presented as the mean ± SEM of three independent experiments performed in triplicate. ***P < 0.001, versus the control. The severity of edema and paw redness was assessed once per day. **c** The degree of paw thickness was detected both in the fore paws and hind paws. **d** Hematoxylin and eosin-stained stained slides were scored blind by a trained observer for immune cell invasion. **e** The depletion of proteoglycans was determined by Safranin O-Fast Green FCF and Toluidine blue staining (100×). The regions of cartilage degeneration, which are light in staining, are marked by arrows

We previously found that E2F2 expression is significantly upregulated in the RA synovium. To determine the role of E2F2 in RA pathogenesis, *E2f2* KO mice were constructed. WT and *E2f2*^−/−^ mice were immunized by a collagen adjuvant mixture. Among *E2f2*^−/−^ mice, 8% (1/12) showed obvious swelling 20 days after the second immunization, and 25% (3/12) showed obvious swelling 50 days after the second immunization. Among WT mice, 58% (7/12) showed obvious swelling 20 days after the second immunization, and 83% (10/12) showed obvious swelling 50 days after the second immunization (Fig. 1a). Meanwhile, the severity of arthritis in WT mice 20 days after the second immunization was significantly higher than in *E2f2*^*−/−*^ mice (Fig. 1b). Edema and erythema/redness in the paws of WT mice was more obvious than in *E2f2*^−/−^ mice at 50 days after the second immunization (Fig. 1c). An immunochemistry assay showed that joint destruction and inflammatory accumulation were significantly decreased in *E2f2*^*−/−*^ mice (Fig. 1d). Next, cartilage degradation was assessed by Safranin O-Fast Green staining, a method for detecting depletion of cartilage. Cartilage loss was significantly decreased in the knee joints of *E2f2*^*−/−*^ mice (Fig. 1e).

### Reduced expression of IL-1α, IL-1β, and TNF-α in E2F2^−/−^ MEFs

**Fig. 2:**
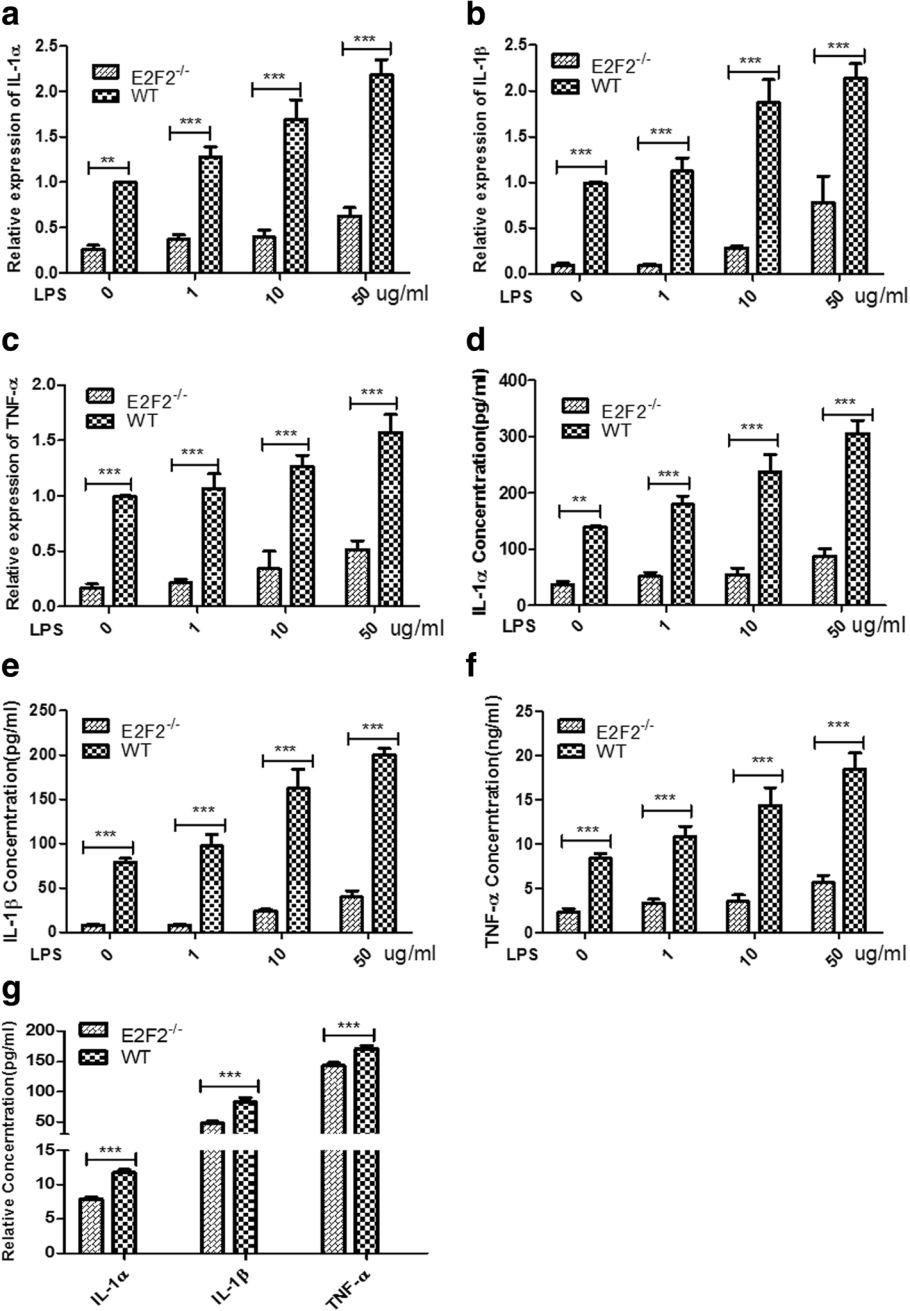
E2F2 is required for production of inflammatory factors in MEFs. MEFs were treated with different concentrations of lipopolysaccharide (LPS) for 12 h. Expression and dose-dependent effects of interleukin (IL)-1α (**a**), IL-1β (**b**), and tumor necrosis factor (TNF)-α (**c**) were detected by qRT-PCR. **P < 0.01, ***P < 0.001, versus vehicle control. Dose-dependent effects of secreted IL-1α (**d**), IL-1β (**e**), and TNF-α (**f**) were detected by ELISA. **P < 0.01, ***P < 0.001, versus vehicle control. IL-1α, IL-1β, and TNF-α in serum of *E2f2*^−/−^ and wild-type (WT) mice were measured by ELISA (**g**). **P < 0.01, ***P < 0.001, versus WT

Inflammatory factors (IL-1α, IL-1β, and TNF-α) play an important role in the progression of RA, and we previously found that siRNAs targeting E2F2 can inhibit the expression of inflammatory cytokines in RASFs [7]. To further verify this result, WT and *E2f2*^−/−^ MEFs were obtained from *E2f2*^+/*−*^ mice and stimulated with LPS for 12 h. qRT-PCR showed that *E2f2* KO significantly inhibited the expression of IL-1α (Fig. 2a), IL-1β (Fig. 2b), and TNF-α (Fig. 2c). ELISA results also confirmed that secretion of IL-1α (Fig. 2d), IL-1β (Fig. 2e), and TNF-α (Fig. 2f) was inhibited in *E2f2* KO MEFs. Secreted IL-1α, IL-1β, and TNF-α was significantly reduced in the blood of the *E2f2*^−/−^ CIA model compared with WT models 60 days after the second immunization (Fig. 2g).

### E2F2 regulates expression of STAT1 and activation of AKT

**Fig. 3:**
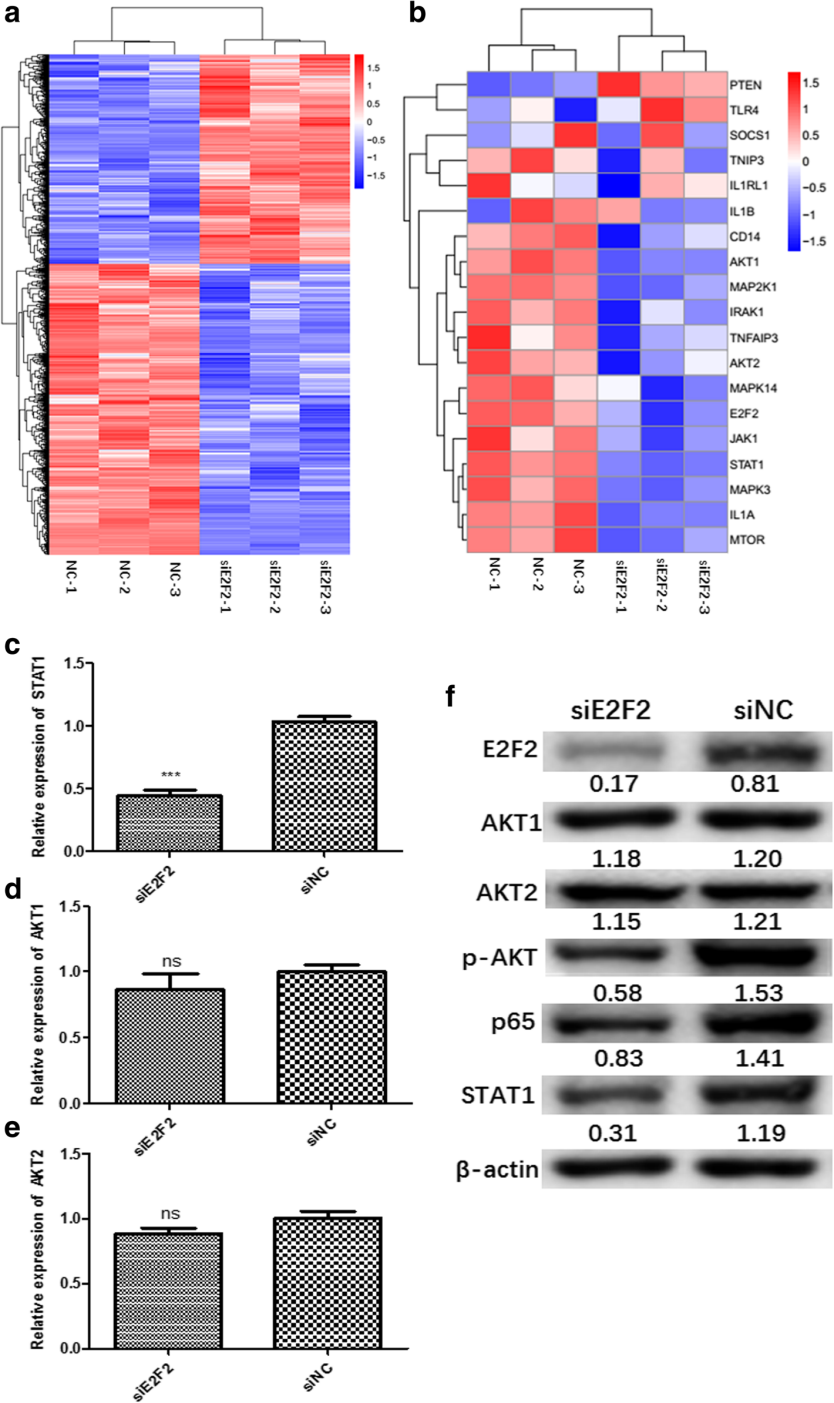
E2F2 participates in RA inflammation through STAT1 and PI3K/AKT/NF-κB pathways. RNA-seq was performed to screen target genes downstream of E2F2 in RASFs. **a**,**b** Heat maps indicate the most differentially expressed genes in RASFs with E2F2 knocked-down. Colored bands represent the change in gene expression: red, downregulation; blue, upregulation. **c**–**e** In-vitro verification of genes related to inflammation in RA was performed using qRT-PCR. mRNA levels of STAT1 (**c**), AKT1 (**d**), and AKT2 (**e**). **f** Western blot was performed to test inhibitory effects of siE2F2 on expression of E2F2, STAT1, AKT1, AKT2, p-AKT, and the p65 subunit of NF-κB. All results are presented as the mean ± SEM of three independent experiments performed in triplicate. NC knockdown scramble control, si small interfering

We further investigated the molecular mechanism by which E2F2 regulates inflammatory cytokines in RA. Control RASFs and their corresponding E2F2 knockdown RASFs from three different RA patients were harvested. RNA-seq was performed to screen target genes downstream of E2F2. Differentially expressed genes following siRNA knockdown of E2F2 were identified (Fig. 3a). qRT-PCR was performed for in-vitro verification. Using KEGG pathway analysis, we found that E2F2 had an obvious effect on two pathways, STAT1 and PI3K/AKT/NF-κB (Fig. 3b). We confirmed this effect using qRT-PCR and Western blot in RASFs and MEFs, and found that E2F2 can affect the phosphorylation activity of AKT but had no significant effect on the expression of AKT1 and AKT2 (Fig. 3c–f).

### E2F2 regulates IL-1α, IL-1β, and TNF-α expression by activating the STAT1 pathway in RASFs and MEFs

**Fig. 4:**
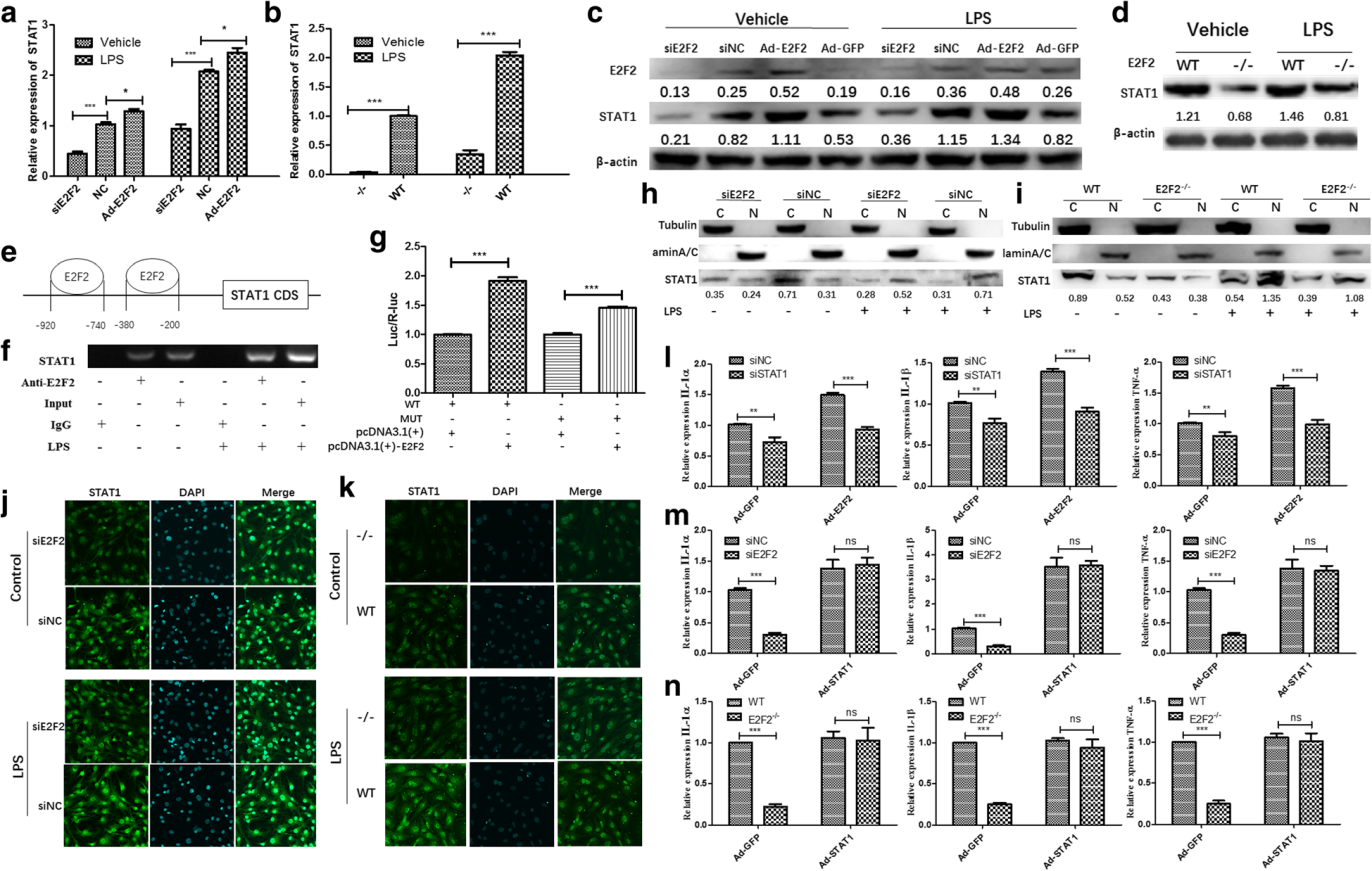
STAT1 mediates E2F2 regulation of interleukin (IL)-1α, IL-1β, and tumor necrosis factor (TNF)-α expression. **a**–**d** Effect of E2F2 on STAT1. E2F2 was overexpressed by adenovirus infection or inhibited by small interfering RNA (siRNA) with or without lipopolysaccharide (LPS; 10 μg/mL). qRT-PCR (**a**,**b**) and Western blot (**c**,**d**) were performed to detect expression of STAT1. **e** Schematic representation of STAT1 promoters, primers for the ChIP assay, and the E2F2 binding motif in the STAT1 promoter. ChIP (**f**) and luciferase (Luc) reporter assays (**g**) were performed to show that E2F2 was recruited to the *STAT1* gene promoter in RASFs in the presence of LPS. Nuclear and cytoplasmic proteins were fractionally extracted from E2F2 knocked-down RASFs (**h**) and *E2f2*^*−/−*^ MEFs (**i**). Effects of E2F2 on nuclear translocation of STAT1 were determined by Western blot. (Lamin A/C as a reference for nuclear extraction (N); Tubulin as a reference for cytoplasmic extraction (C).) Effect of E2F2 on nuclear translocation of STAT1 was observed using confocal fluorescence microscopy both in E2F2-silenced RASFs (**j**) and *E2f2*^*−/−*^ MEFs (**k**). STAT1 (green) was detected using anti-STAT1 antibody. Nuclei were stained with DAPI (blue). **l** In E2F2-overexpressing RASFs, IL-1α, IL-1β, and TNF-α were analyzed by qRT-PCR after silencing STAT1 in the presence of LPS stimulation (10 μg/mL). **m** In STAT1-overexpressing RASFs, IL-1α, IL-1β, and TNF-α were analyzed by qRT-PCR after silencing E2F2 in the presence of LPS stimulation (10 μg/mL). **n** In *E2f2*^*−/−*^ MEFs, expression of IL-1α, IL-1β, and TNF-α was detected using qRT-PCR after STAT1 overexpression in the presence of LPS stimulation (10 μg/mL). The results shown are means ± SEM of three independent experiments performed in triplicate. *P < 0.05, **P < 0.01, ***P < 0.001, versus the control. Ad-GFP adenovirus encoding green fluorescent protein, NC knockdown scramble control, ns not significant, siE2F2 small interfering RNA knockdown of E2F2, WT wild-type

Under LPS stimulation, activated STAT1 proteins can translocate to the cell nucleus and induce transcription of target genes, which may regulate downstream cytokine (IL-1α, IL-1β, TNF-α) production and inflammatory cell infiltration [15, 16]. STAT1, IRF8, and SPI1 can form a complex that binds to the promoter region of IL-1β and promotes its transcription [15]. To test whether E2F2 can directly regulate the expression of STAT1, RASFs were transfected with adenovirus-E2F2 to overexpress E2F2 or siE2F2 to suppress E2F2 expression with or without LPS (10 μg/mL). qRT-PCR and Western blot showed that E2F2 can significantly regulate STAT1 at both the mRNA (Fig. 4a) and protein (Fig. 4c) level. Similarly, mRNA (Fig. 4b) and protein (Fig. 4d) levels of STAT1 in WT MEFs were significantly lower than in *E2f2*^−/−^ MEFs with or without LPS (1 μg/mL).

To further explore the mechanism by which E2F2 regulates STAT1, we found two binding sites of E2F2 on the promoters of STAT1 (Fig. 4e). To validate the binding, ChIP-PCR was performed. The results showed that E2F2 can bind to promoters of STAT1. We can see that LPS treatment (the right three bands) can increase E2F2 binding to STAT1 compared with control (the left three bands).

We also detected the effects of E2F2 on the transcriptional activity of STAT1 by a luciferase activity assay (Fig. 4g), and the results showed that the mutant of binding sites on the promoter of STAT1 can cause a decrease in luciferase activity of E2F2 on STAT1, which indicated that E2F2 can directly bind to the promoter of the *STAT1* gene in RASFs.

STAT1 is also a transcription factor since translocation into the nucleus can result in transcription of target genes [13]. To determine the role of E2F2 in regulating the translocation of STAT1 into the nucleus, we transfected RASFs and MEFs (WT and *E2f2*^−/−^) with siE2F2 in the presence of LPS (10 μg/mL). E2F2 interference significantly decreased the translocation of STAT1 into the nucleus (Fig. 4h, i). Immunofluorescence confirmed the effect of E2F2 on STAT1 translocation in RASFs (Fig. 4j) and MEFs (Fig. 4k).

STAT1 regulates the expression of IL-1α, IL-1β, and TNF-α [14, 16]. To gain further insight into the STAT1-dependent role of E2F2 in the regulation of IL-1α, IL-1β, and TNF-α, STAT1 was silenced by siSTAT1 transfection with or without E2F2 overexpression in RASFs. qRT-PCR showed that inhibition of STAT1 could attenuate the upregulation of IL-1α, IL-1β, and TNF-α (Fig. 4l) by E2F2. Similarly, when E2F2 was silenced in RASFs overexpressing STAT1, the expression of IL-1α, IL-1β, and TNF-α was upregulated (Fig. 4m). Results were confirmed in MEFs; as expected, the expression of the three cytokines decreased in *E2f2*^−/−^ MEFs, but the effect vanished in STAT1-overexpressing MEFs (Fig. 4n).

### E2F2 can regulate the expression of IL-1α, IL-1β, and TNF-α in a STAT1-independent way involving the MyD88-mediated PI3K/AKT/NF-κB pathway

**Fig. 5:**
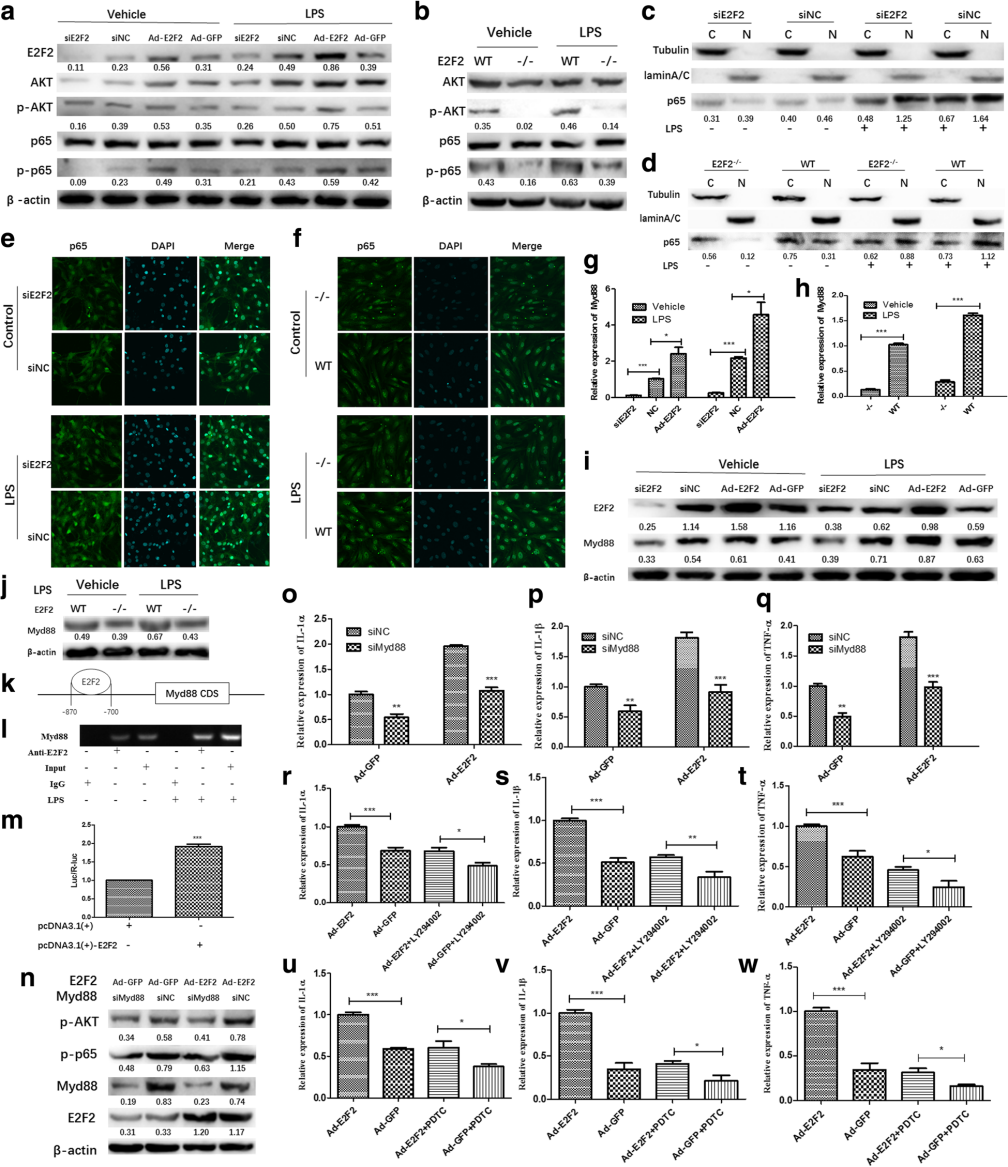
MyD88 mediates regulation of the PI3K/AKT/NF-κB pathway by E2F2. **a**,**b** E2F2 can regulate expression of PI3K/AKT/NF-κB. E2F2-silenced RASFs and *E2f2*^*−/−*^ MEFs were cultured with or without lipopolysaccharide (LPS). Western blot was performed to detect phosphorylation of AKT and NF-κB P65 both in E2F2-silenced RASFs (**a**) and *E2f2*^*−/−*^ MEFs (**b**). **c**,**d** Effect of E2F2 on translocation of p65. E2F2 knocked-down RASFs (**c**) and *E2f2*^*−/−*^ MEFs (**d**) were cultured under LPS stimulation (10 μg/mL) for 12 h; nuclear and cytoplasmic proteins were extracted separately and then Western blot was performed (Lamin A/C as a reference for nuclear extraction (N); Tubulin as a reference for cytoplasmic extraction (C).) **e**,**f** Effects of E2F2 on p65 nuclear translocation both in RASFs (Fig. 4e) and MEFs (Fig. 4f) observed using confocal fluorescence microscopy. **g**–**j** E2F2-silenced RASFs and *E2f2*^*−/−*^ MEFs were cultured with or without LPS. qRT-PCR (**g**,**h**) and Western blot (**i**,**j**) were performed to detect the effect of E2F2 on MyD88. **k** Schematic representation of the MyD88 promoter, primers for the ChIP assay, and the E2F2 binding motif in the MyD88 promoter. **l**,**m** E2F2 was recruited to the *MyD88* gene promoter in RASFs in the presence of LPS. ChIP (**l**) and luciferase (Luc) reporter assay (**m**) were performed in RASFs and MEFs in the presence or absence of LPS (10 μg/mL). **n** MyD88 mediated the effect of E2F2 on PI3K/AKT/NF-κB pathways. Western blot showed that knockdown of MyD88 could significantly inhibit the phosphorylation of AKT and P65 in the presence or absence of E2F2 overexpression. qRT-PCR showed that inhibition of MyD88 can inhibit the expression of inflammatory factors and significantly reduce the upregulation of interleukin (IL)-1α (**o**), IL-1β (**p**), and tumor necrosis factor (TNF)-α (**q**) by E2F2. The effect of inhibitors of PI3K/AKT/NF-κB pathways (LY294002 and PDTC) on E2F2; qRT-PCR was used to detect the effect of inhibitors on expression of IL-1α (**r**,**u**), IL-1β (**s**,**v**), and TNF-α (**t**,**w**) in response to LPS. The results shown are means ± SEM of three independent experiments performed in triplicate. **P < 0.01, ***P < 0.001, versus the control. Ad-GFP adenovirus encoding green fluorescent protein, NC knockdown scramble control, si small interfering, WT wild-type

The PI3K/AKT/NF-κB pathway plays a key role in the regulation of IL-1α, IL-1β, and TNF-α expression in RA [17–21]. We investigated whether E2F2 can affect the expression of IL-1α, IL-1β, and TNF-α through the PI3K/AKT/NF-κB pathway. E2F2-silenced RASFs and *E2f2*^−/−^ MEFs were cultured with or without LPS. Western blot showed that E2F2 could regulate phosphorylation of AKT and NF-κB p65 both in E2F2-silenced RASFs and *E2f2*^−/−^ MEFs, and LPS can increase E2F2 binding to STAT1 (Fig. 5a, b).

To investigate how E2F2 regulates the activation of p65, E2F2 knockdown RASFs and *E2f2*^−/−^ MEFs were used. After stimulation with LPS (10 μg/mL) for 24 h, Western blot was performed with nuclear and cytoplasmic proteins extracted separately. Translocation of p65 into the nucleus was severely inhibited in E2F2 knocked-down RASFs (Fig. 5c) and *E2f2*^−/−^ MEFs (Fig. 5d). Immunofluorescence confirmed the effect of E2F2 on p65 translocation into the nucleus in RASFs (Fig. 5e) and MEFs (Fig. 5f).

Recent studies found that MyD88 can significantly regulate activity of the PI3K/AKT/NF-κB pathway [22, 23]; thus, we investigated whether MyD88 mediates activation of PI3K/AKT/NF-κB by E2F2. We found that E2F2 can directly regulate the expression of MyD88 both at the mRNA (Fig. 5g, h) and protein (Fig. 5i, j) level in E2F2-silenced RASFs and *E2f2*^−/−^ MEFs.

To further explore the mechanism of E2F2 regulation of MyD88, we found one E2F2 binding site in the promoter of MyD88 (using JASPAR 2017 software, http://jaspar.genereg.net/) (Fig. 5k). ChIP-PCR (Fig. 5l) and luciferase reporter assay (Fig. 5m) results suggest that E2F2 can directly bind to the promoter of the *MyD88* gene in RASFs.

To investigate the role of MyD88 in E2F2-regulated inflammatory cytokine expression, E2F2 was overexpressed by adenovirus vector transfection in MyD88-silenced RASFs in the presence of LPS (10 μg/mL). Western blot showed that inhibition of MyD88 could significantly inhibit the phosphorylation of AKT and p65 (Fig. 5n). qRT-PCR showed that inhibition of MyD88 can inhibit the expression of inflammatory factors and significantly attenuated the upregulation of IL-1α (Fig. 5o), IL-1β (Fig. 5p), and TNF-α (Fig. 5q) by E2F2. This was confirmed using inhibitors of the PI3K/AKT/NF-κB pathways (LY294002 and PDTC). qRT-PCR showed that expression of IL-1α (Fig. 5r, u), IL-1β (Fig. 5s, v), and TNF-α (Fig. 5t, w) in response to LPS was significantly attenuated by PDTC and LY294002.

### STAT1/MyD88 complexes mediate the effects of E2F2 on cytokines

**Fig. 6:**
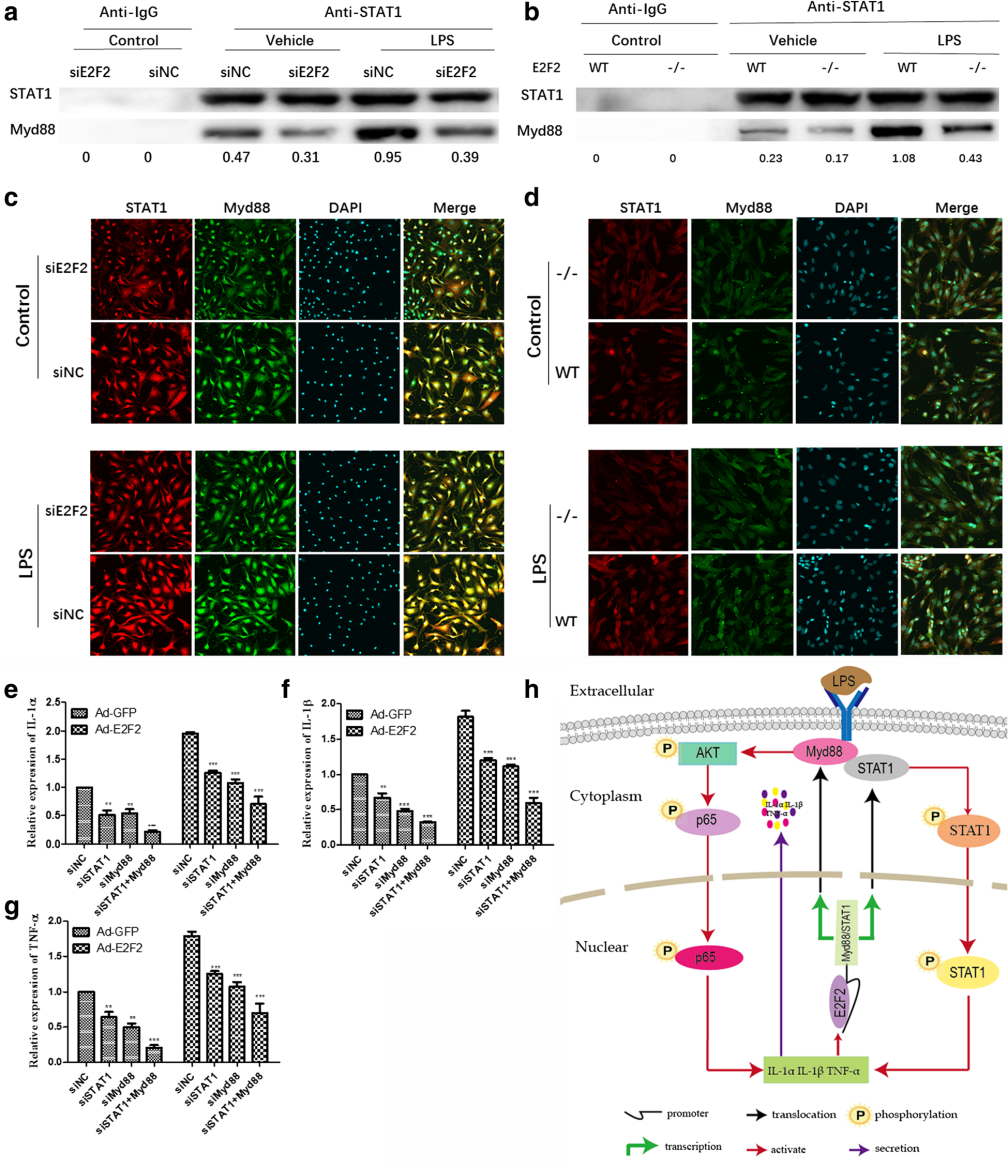
STAT1/MyD88 complexes mediate E2F2 regulation of inflammatory cytokines. **a**–**d** STAT1/MyD88 complex is found in RASFs and MEFs. E2F2-silenced RASFs and *E2f2*^*−/−*^ MEFs were cultured with or without lipopolysaccharide (LPS; 10 μg/mL). Co-IP (**a**,**b**) was performed to test the binding. Confocal immunofluorescence (**c**,**d**) was performed to confirm the result (magnification 10 × 40, MyD88 (green) and STAT1 (red)). **e**–**g** Effect of STAT1/MyD88 complexes on the expression of cytokines. qRT-PCR was performed to detect expression of interleukin (IL)-1α (**e**), IL-1β (**f**), and tumor necrosis factor (TNF)-α (**g**) in STAT1/MyD88 knockdown RASFs with or without E2F2 overexpression in the presence of LPS (10 μg/mL). The results shown are means ± SEM of three independent experiments performed in triplicate. **P < 0.01, ***P < 0.001, versus the control. **h** Model depicting the role of E2F2 in RA pathogenesis. Ad-GFP adenovirus encoding green fluorescent protein, NC knockdown scramble control, si small interfering, WT wild-type

A number of studies have shown that MyD88 directly controls activation of the PI3K/AKT/NF-κB pathway and regulation of inflammation in murine macrophages [24], and that STAT1 can promote the progression of RA [16, 25, 26]. Moreover, the STAT1/MyD88 complex mediates activation of STAT1 [27]. We hypothesized that STAT1/MyD88 complexes might mediate the effects of E2F2 on IL-1α, IL-1β, and TNF-α. To verify our hypothesis, co-IP and immunofluorescence assays were performed to detect binding of STAT1 with MyD88 both in E2F2-silenced RASFs and *E2f2*^−/−^ MEFs. As expected, both co-IP (Fig. 6a, b) and immunofluorescence assays (Fig. 6c, d) showed that inhibition of E2F2 can significantly inhibit the formation of MyD88 and STAT1 complexes, and in turn affect the activation of STAT1 and AKT and ultimately regulate the expression of IL-1α, IL-1β, and TNF-α. MyD88, STAT1, and MyD88/STAT1 were knocked-down by siRNA with or without overexpression of E2F2 under stimulation with LPS (10 μg/mL). qRT-PCR showed that inhibition of both MyD88 and STAT1 could significantly attenuate the E2F2-stimulated expression of IL-1α (Fig. 6e), IL-1β (Fig. 6f), and TNF-α (Fig. 6g).

## Discussion

Hyperplasia and excessive production of inflammatory factors in RASFs leads to joint damage, and RASFs play a central role in RA pathogenesis [26]. Previous studies have shown that inhibition of E2Fs by oligodeoxynucleotides can prevent cartilage destruction by inhibition of synovial cell proliferation [8, 28]. Our previous microarray assay and the following studies showed that E2F2 expression is significantly higher in RA synovial tissue than in osteoarthritis (OA), and that E2F2 is associated with the pathological progression of RA and can exacerbate inflammatory phenotypes in RASFs, such as proliferation, invasion, and cytokine production in vitro [7]. In the present study, we investigated the mechanism of E2F2 in RA inflammation.

We developed *E2f2*^−/−^ mice to test the role of E2F2 in promoting inflammation. As previously reported by Murga et al. [11], *E2f2*^−/−^mice survive to adulthood, are fertile, produce normal offspring, and have normal gross and microscopic organ morphology at 4- to 8-weeks of age. However, only 27% of the E2F2-deficient animals survive to 15 months. When developing our CIA models, 8-week-old mice were used. We did not observe any significant differences in the blood routine index in WT and *E2f2*^−/−^ mice. In *E2f2*^−/−^ mice, IL-1α, IL-1β, and TNF-α expression in MEFs and secretion in the serum were significantly decreased. CIA was induced following traditional methods. After 2 months, *E2f2*^−/−^ mice had significantly reduced progression of CIA, both in incidence, severity, and cartilage destruction. We also aimed to uncover the mechanism by which E2F2 acts in RA inflammation. Indeed, we found that E2F2 can regulate the expression of STAT1 and activation of the PI3K/AKT/NF-κB pathway.

STAT1 is a member of the STAT family of transcription factors. STAT1 expression was reported to be significantly higher in RA synovial tissue than in OA and mandatory spondylitis [3]. In addition, functionalized STAT1 siRNA nanoparticles can regress CIA in a mouse model [29]. Notably, STAT1 regulates the expression of IL-1α, IL-1β, and TNF-α in THP-1 cells and RAW 264.7 cells [16, 30]. We tested whether STAT1 can also regulate the expression of these three cytokines in RASFs and MEFs. To investigate how E2F2 regulates STAT1, we performed co-IP and luciferase reporter assays. We found that E2F2 can bind to the promoter of STAT1 and regulate its expression. Expression of E2F2 significantly induced translocation of STAT1 into the nucleus and subsequently regulated the expression of IL-1α, IL-1β, and TNF-α. To further verify this, we knocked-down STAT1 in normal or E2F2-overexpressing RASFs and found that, in normal RASFs, siSTAT1 reduced the LPS-stimulated inflammatory cytokines by 25% while, in E2F2-overexpressing RASFs, siSTAT1 reduced the LPS-stimulated inflammatory cytokines by 50%. On the other hand, E2F2 knockdown can result in decreased STAT1 and LPS-stimulated inflammatory cytokines. Recovery of STAT1 prevented the inhibition of cytokine expression by siE2F2 and in *E2f2*^−/−^ MEFs. Thus, it is important to explain how E2F2 regulates the expression of inflammatory cytokines. We inferred that E2F2 may play a role in regulation of RA inflammation through the STAT1 pathway. However, inhibition of STAT1 activity does not completely reverse the upregulation of IL-1α, IL-1β, and TNF-α by E2F2, suggesting that E2F2 may have other pathogenic pathways. Therefore, we tried to find other pathways that participate in the effect of E2F2 in RA.

Using KEGG pathway analysis, we found that E2F2 had a significant effect on the PI3K/AKT/NF-κB pathway. The PI3K/AKT/NF-κB pathway regulates the immune response by provoking production of the cytokines IL-1α, IL-1β, and TNF-α in RASFs [21]. We demonstrated that E2F2 can significantly regulate the activation of the PI3K/AKT/NF-κB pathway and improve the translocation of p65 (a subunit of NF-κB) into the nucleus. We next tried to find a mediator between E2F2 and PI3K/AKT/NF-κB. From the literature, we found that MyD88, an adaptor protein for Toll-like receptor (TLR)4, can regulate expression of IL-1α, IL-1β, and TNF-α depending on activation of PI3K/AKT/NF-κB in RASFs [22, 31]. Notably, we further observed that E2F2 can bind to the promoter of the *MYD88* gene and directly regulate the expression of MyD88 both at the mRNA and protein level. Thus, MyD88 may be the mediator linking E2F2 to cytokines. To test this, we knocked-down MYD88 in RASFs, and found that inhibition of MYD88 may inactivate AKT and p65 and inhibit cytokine expression both in normal and E2F2-overexpressing RASFs. Specific inhibitors of PI3K/AKT and NF-κB were used to confirm the involvement of these pathways. Our results suggest that E2F2 can regulate expression of cytokines at the mRNA level via the PI3K/AKT/NF-κB pathway in RASFs. STAT1 can bind to MyD88 and the bond may affect activation of STAT1 [23]. Co-IP and immunofluorescence assays of RASFs and MEFs consistently confirmed the bond between STAT1 and MyD88. Furthermore, our data suggest that simultaneous knockdown of STAT1 and MyD88 could decrease the expression of inflammatory factors more significantly than inhibition of one of them alone, and the trend is more pronounced in E2F2-overexpressing RASFs. These results indicate that E2F2 can positively regulate the expression of STAT1/MyD88 in RASFs and MEFs, which in turn may play a role in mediating the expression of these proinflammatory cytokines. Therefore, there may be an E2F2-STAT1/MyD88-cytokine loop in the in vivo inflammatory microenvironment.

## Conclusion

In summary, our study sheds light on the proinflammatory role of E2F2 in the pathogenesis of RA (Fig. 6h). E2F2 affects the formation of the STAT1/MYD88 complex by directly binding to the STAT1 and MYD88 promoters. This in turn influences the entry of STAT1 into the nucleus and activation of the PI3K/AKT/NF-ΚB pathway, ultimately regulating expression of inflammatory cytokines including IL-1α, IL-1β, and TNF-α. A better understanding of E2F2-mediated inflammatory pathways may lead to novel treatment strategies for RA.

## Abbreviations

ChIP: Chromatin immunoprecipitation
CIA: Collagen-induced arthritis
Co-IP: Co-immunoprecipitation
E2F2: E2F transcription factor 2
ELISA: Enzyme-linked immunosorbent assay
IL: Interleukin
KO: Knockout
LPS: Lipopolysaccharide
MEF: Mouse embryonic fibroblast
Mut: Mutant
MyD88: Myeloid differentiation primary response 88
OA: Osteoarthritis
PBS: Phosphate-buffered saline
PCR: Polymerase chain reaction
qRT-PCR: Quantitative real-time polymerase chain reaction
RA: Rheumatoid arthritis
RASF: Rheumatoid arthritis synovial fibroblast
siRNA: Small interfering RNA
STAT1: Signal transducer and activator of transcription 1
TNF: Tumor necrosis factor
WT: Wild-type
